# Effect of physical activity on reducing the risk of diabetic retinopathy progression: 10-year prospective findings from the 45 and Up Study

**DOI:** 10.1371/journal.pone.0239214

**Published:** 2021-01-14

**Authors:** Xixi Yan, Xiaotong Han, Changfan Wu, Xianwen Shang, Lei Zhang, Mingguang He

**Affiliations:** 1 Eye Center, Renmin Hospital of Wuhan University, Eye Institute of Wuhan University, Wuhan, China; 2 Centre for Eye Research Australia, Ophthalmology, Department of Surgery, Royal Victorian Eye and Ear Hospital, University of Melbourne, Melbourne, Australia; 3 State Key Laboratory of Ophthalmology, Zhongshan Ophthalmic Center, Sun Yat-sen University, Guangzhou, China; 4 Department of Ophthalmology, Yijishan Hospital of Wannan Medical College, Wuhu, China; 5 Melbourne Sexual Health Centre, Alfred Health, Melbourne, Australia; 6 Faculty of Medicine, Central Clinical School, Monash University, Melbourne, Australia; 7 Department of Epidemiology and Biostatistics, School of Public Health, Xi’an Jiaotong University Health Science Center, Xi’an, Shaanxi, China; University of Maiduguri College of Medical Sciences, NIGERIA

## Abstract

**Objective:**

To examine the association of physical activities (PA) with diabetic retinopathy (DR) progression based on a 10-year follow-up of a large cohort of working-aged diabetic populations in Australia.

**Methods:**

Nine thousand and eighteen working-aged diabetic patients were enrolled from the baseline of the 45 and Up Study from New South Wales, Australia. Self-reported PA collected by questionnaire at baseline in 2006 was graded into low (<5 sessions/week), medium (≥5–14), and high (≥14) levels. Retinal photocoagulation (RPC) treatment during the follow-up period was used as a surrogate for DR progression and was tracked through the Medicare Benefits Schedule, which was available from 2004 to 2016. Cox regression was used to estimate the association between PA and RPC incidence.

**Results:**

In the fully adjusted model, higher PA level was significantly associated with a lower risk of RPC incident (Cox-regression, p-value for trend = 0.002; medium vs. low, hazard ratio (HR) = 0.78, 95% Confidence Interval (CI): 0.61–0.98; high vs. low, HR = 0.61, 95%CI: 0.36–0.84. In addition, gender, body mass index, insulin treatment, family history of diabetes, history of cardiovascular disease were significant effect modifiers for the association between PA and RPC.

**Conclusions:**

Higher PA level was independently associated with a lower risk of DR progression among working-aged diabetic populations in this large cohort study.

## Introduction

Diabetes has become a public health burden worldwide, with an estimated prevalence of 9% (accumulated 642 million patients) by 2040 [1]. As one of the most common microvascular complication of diabetes, diabetic retinopathy (DR) is the leading cause of visual impairment and blindness in the working-aged population [2]. To date, there are about 100 million DR patients worldwide, and one third suffer vision-threatening DR [3]. These numbers are projected to grow in the following decades with the increasing diabetes populations, resulting in a tremendous burden on the economic and health system.

Physical activity (PA), as a modifiable risk factor, has been well established as a treatment strategy for diabetes, but its effect on preventing DR and its progression has not yet been proven. Several studies have investigated this association, but the findings are inconsistent, compromised by relatively small sample size and short follow up period. No associations were found in type 1 diabetes patients in the Pittsburgh study [4], and a protective effect of PA on DR risk was observed in women only in the WESDR study [5]. Studies conducted in type 2 diabetic patients found that higher levels of PA were associated with milder DR and less likelihood of DR onset; these studies were cross-sectional in design or limited by small sample size [6–8]. There is only one longitudinal study recently published, reporting a lower DR incidence in participants with higher PA levels, but this study was conducted in a small sample of the cohort during 2-year follow-up [9]. To the best of our knowledge, the longitudinal relationship between PA and progression to a vision-threatening stage of DR has not been reported.

Severe non-proliferative diabetic retinopathy (sNPDR) and proliferative diabetic retinopathy (PDR), regardless of the presence of macular edema, are usually vision threatening [10] and are considered as an indication for retinal photocoagulation (RPC) treatment [11]. Thus, RPC could be utilized as a surrogate for DR progression into a more severe stage. The purpose of the present study was to investigate the relationship between PA and DR progression among working-age diabetic patients over eight years based on a large Australian cohort.

## Materials and methods

### Study population

The Sax Institutes’ 45 and Up Study is the largest prospective cohort study in Australia [12]. This study enrolled 266,896 residents aged 45 years and older in the state of New South Wales (NSW), Australia at baseline from 2006 to 2009, representing an estimated 18% response rate and around 10% of the NSW population in this age group [12]. Eligible participants were randomly sampled from the Department of Human Services (formerly Medicare Australia) enrolment database and received a mailed invitation including a study questionnaire and a written informed consent form (including consent for linkage of their data to other population health databases). The baseline questionnaire captured information on a broad range of socioeconomic status, health conditions, and health-related lifestyles. The study methodology had been described in detail elsewhere [12], and the baseline questionnaire is available at http://www.saxinstitute.org.au/our-work/45-up-study/questionnaires/. The 45 and Up Study was linked to the Medicare Benefits Schedule (MBS) and Pharmaceutical Benefits Scheme (PBS) in order to track the procedures and medications that the participants had claimed. Both MBS and PBS are the core of Australia’s universal healthcare system, the MBS data are generated by financial reimbursement claims for diagnosed test and treatment from General Practitioners, Specialists, Allied Health, and Hospitals, while the PBS data generated by claims for the drug from pharmacies and other health services. Linkage to the 45 and Up Study Cohort is done by the Sax Institute using a unique identifier that was provided by the Department of Human Services. The MBS data are available from 24 January 2001 to 31 December 2016, and the PBS data from 1 June 2004 to 31 December 2016. Thus, any procedure of participants received from the baseline survey to the end of 2016 could be tracked. The 45 and Up Study was approved by the ethical board of the University of NSW Human Research Ethics Committee and was conducted in accordance with the tenets of the Declaration of Helsinki. Approval to use data from the 45 and Up Study for the current study was received from the Center for Eye Research Australia Ethics Committee.

**Fig 1:**
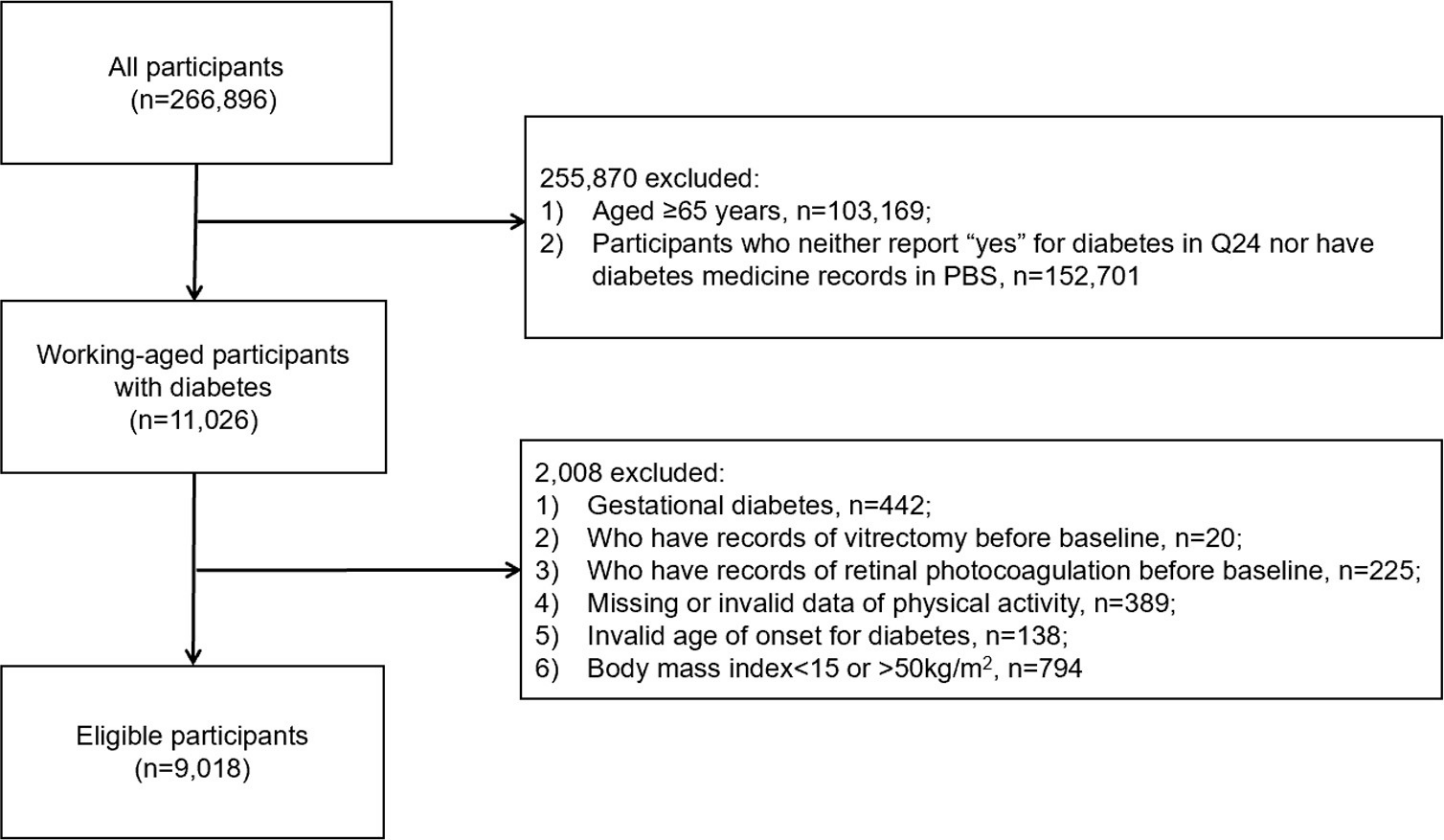
Algorithm for identifying eligible participants.

The current study only included working-age participants with diabetes from the 45 and Up Study at baseline, and detailed inclusion criteria were as follows: (1) positive response to question No.24 ‘Has a doctor EVER told you that you have diabetes?’ or recorded use of diabetes medications based on PBS database, which had been proved to be a satisfied complementary to identify a diabetic patient [13]. (2) aged 45–65 years. Exclusion criteria included: (1) gestational diabetes, defined as a diagnosis of diabetes earlier than the last childbirth, but without antihyperglycemic medication record afterwards; (2) history of vitrectomy or RPC based on MBS data before baseline; (3) missing data or invalid data (outliers) of PA; (4) invalid age for diabetes onset: reported age at diabetes diagnosis older than the age at baseline survey; (5) Body mass index (BMI) data were considered not reliable such as reported as <15kg/m^2^, or >50 kg/m^2^. Fig 1 shows the process of participant selection.

### Variables

#### Outcome

The main outcome is whether baseline participants received RPC (includes both pan-retinal photocoagulation and focal retinal treatment for macular edema) during the follow-up period, which is an indicator of progression to sNPDR or PDR. A participant who had at least one retinal laser treatment record in the MBS database (MBS code for RPC: 42809) were defined as had undergone RPC. The date of first laser treatment was utilized for analysis if the patient underwent multiple RPC treatments.

#### Physical activity

Items from the Active Australia Survey (AAS) [14] was used to evaluate PA at baseline in the 45 and Up Study’s baseline questionnaire with proved good test-retest reliability [15] and validity [16]. In the questionnaire, a session of PA was defined as follows: walking continuously for at least 10 min (for recreation or exercise or to get to or from places), moderate PA (e.g., gentle swimming, social tennis, vigorous gardening, or work around the house), and vigorous PA (that made you breathe harder or puff and pant, such as jogging, cycling, aerobics, and competitive tennis but not household chores or gardening). The metabolic equivalent (MET) intensity level number of weekly sessions of PA was calculated for all participants. The computational formula is W + M + 2V, wherein W represents the number of times of walking, M represents times of moderate activity, and V represents times of vigorous activities [17]. Participants were classified into three PA categories based on the above MET‐adjusted session: <5 sessions/week (low), ≥5–14 sessions/week (medium), and ≥14 sessions/week (high).

#### Covariates

Covariates included age, gender, educational level, household income per year, BMI, history of hypertension and cardiovascular disease (CVD), family history of diabetes, insulin treatment, duration of diabetes, smoking status, and alcohol drinking status at baseline. All the covariates except for insulin treatment were derived from the baseline questionnaire while the insulin treatment records were derived from available PBS database. BMI was calculated as the self-reported weight in kilograms divided by the square of self-reported height in meters, which had been reported in excellent agreement with objectively measured BMI categories in a subsample of 45 and Up Study [18]. Based on the World Health Organization criteria, BMI was divided into underweight (<18.5kg/m^2^), normal (18.5–24.9 kg/m^2^), overweight (25–29.9 kg/m^2^), and obese (≥30.0 kg/m^2^). History of CVD was defined as who has any history of stroke or heart disease history indicated in Q24 of the baseline questionnaire. The duration of diabetes was calculated as the age at baseline minus the age at the diagnosis of diabetes for participants enrolled based on the questionnaire, while for participants enrolled based on PBS database, the duration was calculated as the age at baseline minus the age of the first prescription of any antihyperglycemic medication. Duration of diabetes was further divided into four groups: 0–5, ≥5–10, ≥10–20, ≥20 years. Insulin treatment diabetes was defined as those who had insulin medication record in the PBS during the follow-up period.

### Statistical analysis

The t-test was used to test the difference of continuous variables between participants who did and did not undergo RPC treatment. One-way ANOVA for continuous variables and *χ*^2^ test for categorical variables were used to examine the difference in baseline characteristics between PA groups. The association between PA categories and RPC incidence were analyzed using the Cox proportional hazards model. The hazard ratio (HR; 95% CIs) was estimated for the outcome in comparison with a reference category of patients with a PA of <5 MET-sessions/week. We tested the following models: 1) crude model, calculate separate HRs for variables including age, gender, household income, education, BMI, history of hypertension, CVD, family history of diabetes, insulin treatment, diabetes duration, current smoker, alcohol drinking and PA; 2) adjusted model, adjusted for all variables in the crude model.

We also investigated the association between PA and RPC in subgroups stratified by gender, BMI, insulin treatment, family history of diabetes, history of hypertension and CVD. All p values were two-sided, and a p-value of <0.05 was considered statistically significant. All analyses were performed using SAS (version 9.4; SAS Institute, Inc).

## Results

### Demographic characteristics

A total of 9018 eligible diabetic participants were included in this study, with a mean age of 57.2±5.0 years, and 43.9% were female. Overall, the mean age at diabetes diagnosis was 49.3±9.3 years, and 85.5% of the participants were overweight or obese. Over two-thirds (68.4%) of the participants had a history of diabetes for less than ten years. The proportions of participants with a PA level of <5, ≥5–14, ≥14 sessions per week were 26.2%, 51.9% and 21.9%, respectively. Baseline characteristics of participants were presented in Table 1, stratified by PA level and RPC status during the follow-up period. The PA level was significantly lower in women (*χ*^2^ test, p = 0.005), lower education level (p<0.001), obesity (p = 0.005), a history of hypertension (p = 0.012) or CVD (p<0.001), insulin treatment (p<0.001) and non-drinker (p<0.001). Significantly higher proportions of RPC were found in patients with a family history of diabetes (*χ*^2^ test, p = 0.002), longer diabetes duration (p<0.001), insulin treatment (p<0.001), nondrinker (p = 0.009) and lower level of PA (p = 0.006), compared to otherwise.

**Table 1:** Comparison of baseline characteristics among participants with different physical activity levels and between participants who did and did not receive retinal photocoagulation during the follow-up period.

Characteristics	All n = 9018	Physical activity (sessions/week)			p-value*	Retinal photocoagulation		p-value*
		<5 n = 2364	5–14 n = 4680	≥14 n = 1974		Yes n = 364	No n = 8654	
Age (years)					0.68			0.22
45–55	2917(32.3)	804(34.0)	1448(30.9)	665(33.7)		107(29.4)	2810(32.5)	
56–65	6101(67.7)	1560(66.0)	3232(69.1)	1309(66.3)		257(70.6)	5844(67.5)	
Female	3960(43.9)	1050(44.4)	2122(45.3)	788(39.9)	0.005	160(44.0)	3800(43.9)	0.99
Household income (AUD/year) †					0.32			0.88
<20000	2028(22.5)	561(23.7)	1065(22.8)	402(20.4)		83(22.8)	1945(22.5)	
≥20000–40000	1495(16.6)	375(15.9)	772(16.5)	348(17.6)		62(17.0)	1433(16.6)	
≥40000–70000	1735(19.2)	445(18.8)	892(19.1)	398(20.2)		71(19.5)	1664(19.2)	
≥70000	2141(23.7)	549(23.2)	1118(23.9)	474(24.0)		79(21.7)	2062(23.8)	
Education †					<0.001			0.36
Low ‡	1270(14.1)	390(16.5)	649(13.9)	231(11.7)		52(14.3)	1218(14.1)	
Medium ‡	5822(64.6)	1516(64.1)	3061(65.4)	1245(63.1)		243(66.8)	5579(64.5)	
High ‡	1835(20.3)	429(18.1)	933(19.9)	473(24.0)		68(18.7)	1767(20.4)	
Body mass index (kg/m^2^)					<0.001			0.47
Underweight and normal weight	1307(14.5)	209(8.8)	722(15.4)	376(19.0)		57(15.7)	1250(14.4)	
Overweight	2960(32.8)	648(27.4)	1573(33.6)	739(37.4)		121(33.2)	2839(32.8)	
Obese	4751(52.7)	1507(63.7)	2385(51.0)	859(43.5)		186(51.1)	4565(52.8)	
Hypertension	5328(59.1)	1457(61.6)	2725(58.2)	1146(58.1)	0.01	232(63.7)	5096(58.9)	0.07
Cardiovascular disease	1667(18.5)	498(21.1)	834(17.8)	335(17.0)	<0.001	66(18.1)	1601(18.5)	0.86
Family history of diabetes	4769(52.9)	1219(51.6)	2530(54.1)	1020(51.7)	0.83	221(60.7)	4548(52.6)	0.002
Insulin treatment	1237(13.7)	356(15.1)	627(13.4)	254(12.9)	<0.001	113(31.0)	1124(13.0)	<0.001
Diabetes duration (years) †					0.10			<0.001
<5	3938(43.7)	1027(43.4)	2021(43.2)	890(45.1)		82(22.5)	3856(44.6)	
≥5–10	2227(24.7)	537(22.7)	1207(25.8)	483(24.5)		78(21.4)	2149(24.8)	
≥10–20	1868(20.7)	529(22.4)	958(20.5)	381(19.3)		124(34.1)	1744(20.2)	
≥20	590(6.5)	169(7.1)	293(6.3)	128(6.5)		66(18.1)	524(6.1)	
Current smoker	973(10.8)	288(12.2)	477(10.2)	208(10.5)	0.14	25(6.9)	948(11.0)	0.12
Alcohol drinker	4839(53.7)	1115(47.2)	2582(55.2)	1142(57.9)	<0.001	176(47.8)	4663(53.9)	0.009
Physical activity (sessions/week)								0.006
<5	2364(26.2)	-	-	-	-	118(32.4)	2246(26.0)	
≥5–14	4680(51.9)	-	-	-		185(50.9)	4495(52.0)	
≥14	1974(21.9)	-	-	-		61(16.8)	1913(22.1)	

Values are all listed as n (%).

* p-value for comparing the composed rate of baseline characteristics by χ^2^ test.

† Proportion of missing value for household income, education and diabetes duration was 18.0%, 1.0% and 4.4%, respectively.

‡ Low, no qualifications; medium, certificate/diploma/trade; high, university.

### Association between physical activity and retinal photocoagulation

During a mean follow-up period of 8.4 years, 364 (4.0% of participants) participants received RPC. Table 2 shows the results from the Cox proportional hazards regression analysis of the relationship between baseline characteristics and RPC. In the fully adjusted model, when compared to those who reported a low level (<5 sessions/week) of PA, the hazards ratios (95%CI) for RPC were 0.78(0.61–0.98) and 0.61 (0.36–0.84) for medium level (≥5–14 sessions/week) and high level (≥14 sessions/week) of PA, respectively (p-value for trend = 0.002). Participants who had ever been treated with insulin (HR = 2.89, 2.30–3.62) and with longer duration of diabetes (p-value for trend <0.001) had a higher risk of RPC.

**Table 2:** Cox regression analysis of risk factors for RPC in diabetic patients.

Factors	Event/N	Crude model		Adjusted model§	
		HR (95% CI)	p-value	HR (95% CI)	p-value
Age (years)			0.23		-
45–55 (Ref.)	107/2917	1		-	
56–65	257/6101	1.15(0.92–1.44)		-	
Female (Ref. male)	160/3960	1.01(0.82–1.24)	0.95	-	-
Household income (AUD/year) †			0.53		-
<20000 (Ref.)	83/2028	1		-	
≥20000–40000	62/1495	1.01(0.73–1.41)		-	
≥40000–70000	71/1735	1.00(0.73–1.37)		-	
≥70000	79/2141	0.90(0.66–1.23)		-	
Education †			0.53		-
Low ‡ (Ref.)	52/1270	1		-	
Medium ‡	243/5822	1.02(0.76–1.38)		-	
High ‡	68/1835	0.91(0.63–1.30)		-	
BMI			0.47		-
Underweight and normal weight (Ref.)	57/1370	1		-	
Overweight	121/2960	0.94(0.68–1.28)		-	
Obese	186/4751	0.90(0.67–1.21)		-	
Hypertension (Ref. No)	232/5328	1.22(0.99–1.51)	0.07	-	-
Cardiovascular disease (Ref. No)	66/1667	0.97(0.75–1.27)	0.85	-	-
Family history of diabetes (Ref. No)	221/4769	1.39(1.12–1.71)	0.002	-	-
Insulin treatment (Ref. No)	113/1237	3.04(2.43–3.80)	<0.001	2.89(2.30–3.62)	<0.001
Diabetes duration (years) †			<0.001		<0.001
<5 (Ref.)	82/3938	1		1	
≥5–10	78/2227	1.69(1.24–2.31)		1.58(1.15–2.15)	
≥10–20	124/1868	3.29(2.49–4.34)		2.67(2.00–3.56)	
≥20	66/590	5.68(4.11–7.85)		4.09(2.87–5.83)	
Current smoker (Ref. No)	25/976	0.60(0.40–0.90)	0.01	-	-
Alcohol drinker (Ref. No)	176/4839	0.83(0.67–1.03)	0.09	-	-
Physical activity (sessions/week)			0.001		0.002
<5 (Ref.)	118/2364	1		1	
≥5–14	185/4680	0.79(0.62–0.99)		0.78(0.61–0.98)	
≥14	61/1974	0.61(0.45–0.83)		0.61(0.36–0.84)	

Abbreviation: HR, hazard ratios; CI, confidence interval; RPC, retinal photocoagulation; BMI, body mass index.

† Proportion of missing value for household income, education and diabetes duration was 18.0%, 1.0% and 4.4%, respectively.

‡ Low, no qualifications; medium, certificate/diploma/trade; high, university.

§ Adjusted for all the factors in crude models.

### Subgroup analysis for associations between physical activity and retinal photocoagulation

Analysis of interaction effect showed the relation of PA to DR progression stratified by several potential effect modifiers (Table 3). When low-level PA was used as a reference, significant associations between PA and RPC was found only in men (p-value for trend <0.001), overweight participants (p-value for trend = 0.01), those who did not use insulin (p-value for trend <0.001), those with a family history of diabetes (p-value for trend = 0.003), and those without CVD (p-value for trend <0.001). Gender (p-value for interaction = 0.03), BMI (p-value for interaction = 0.004), insulin treatment (p-value for interaction<0.001), family history of diabetes (p-value for interaction<0.001) and CVD (p-value for interaction<0.001) were all significant modifiers for associations between PA and RPC.

**Table 3:** Fully adjusted models for associations between physical activity and retinal photocoagulation stratified by gender, BMI, insulin used, family history of diabetes, hypertension and cardiovascular disease †.

Factors	Physical activity (sessions/week)			
	5–14 versus <5	≥14 versus <5	p-value for trend	p-value for interaction
	HR (95% CI)	HR (95% CI)		
Gender				0.03
Men	0.59(0.43–0.80)	0.43(0.28–0.65)	<0.001	
Women	1.10(0.75–1.60)	0.96(0.59–1.56)	0.88	
BMI				0.004
Underweight and normal weight	0.69(0.36–1.33)	0.45(0.20–1.00)	0.05	
Overweight	0.77(0.51–1.17)	0.49(0.28–0.86)	0.01	
Obese	0.77(0.56–1.06)	0.76(0.49–1.16)	0.12	
Insulin treatment				<0.001
Yes	0.82(0.54–1.26)	0.89(0.52–1.52)	0.59	
No	0.76(0.58–1.01)	0.52(0.35–0.76)	<0.001	
Family history of diabetes				0.003
Yes	0.79(0.59–1.07)	0.54(0.36–0.83)	0.003	
No	0.75(0.51–1.08)	0.70(0.43–1.12)	0.10	
Cardiovascular disease				<0.001
Yes	0.60(0.34–1.05)	0.96(0.50–1.82)	0.66	
No	0.81(0.62–1.05)	0.53(0.37–0.76)	<0.001	

Abbreviation: BMI, body mass index; HR, hazard ratios; CI, confidence interval.

† Adjusted for all the factors in crude models.

## Discussion

In this large cohort of working-aged Australians with diabetes, a higher level of PA was an independent protective factor for DR progressing to severe stages which requires RPC treatment. Interestingly, our study found that this protective effect was of dose-response, the people who had ≥14 PA sessions per week would further reduce the risk on developing severe DR progression in comparison with those had 5–14 sessions per week. Furthermore, the increased PA time appears to be effective only among people with mild diabetes, such as those who are not on insulin treatment, or those without established cardiovascular diseases.

Most previous studies reported associations between PA and DR in the middle-aged and elderly diabetic population were cross-sectional in design and therefore could not establish causal effects. Anna et al. found a significant negative correlation between PA and DR severity in a cross-sectional study (HR = 0.73, 0.66–0.80; p<0.05) [7]. However, another cross-sectional study from Indonesian reported that PA was not correlated to vision-threatening DR, but had a protective effect on nephropathy, which is another common microvascular complication of diabetes [6]. Nevertheless, a recent longitudinal study in Japan showed that higher levels of PA were independently associated with a lower incidence of DR in patients with type 2 diabetes (HR = 0.63, 0.42–0.94; p<0.05) but this analysis was based on 2-years follow up data on 1,814 participants [9].

Although PA has been identified as the most important modifiable risk factor for diabetic control, its pathophysiologic mechanisms in the prevention of DR onset and progression was not fully understood. Reported key pathological contributors in the development of DR include hemodynamic changes, oxidative stress and so on [19], Emmanuel et al. found that PA could improve hemodynamic parameters in a healthy population [20], and R. Elosua et al. found regular PA played a positive role in favorably modifying the antioxidant–prooxidant balance [21]. Other systemic benefits from PA include glycemic improvement, increase in insulin sensitivity, and maintain of endothelial function [22]. Also, PA could decrease the risk of hyperlipidemia and hypertension [23], which are also systemic risk factors for DR.

We found that risk reductions in DR progression through PA were significant in men only. The Pittsburgh study reported that male diabetes who participated in team sports were less likely to report macrovascular disease during follow-up than otherwise [4], while an earlier study found significant associations between strenuous activities and DR incidence in females only [5]. Possible explanations for the gender difference may include different strenuous type and amount of activities performed by females and males. In the interaction analysis, the association between PA and RPC was borderline significant in under to normal-weight participants and significant in overweight participants, but not in obese individuals. These may due to less proportion of vigorous PA by physical function limitation caused by obesity [24]. The effects of PA on DR progression are substantially stronger in participants who do not require insulin treatment, which is consistent with previous findings [4,25]. This may be due to the effect of PA on glycaemic control, blood pressure control and beta-cell function protection, which had been well proved in type 2 diabetic patients [26], but until now, there is no evidence for the protective role of PA in type 1 diabetic patients who require insulin treatment. The significant beneficial effect of PA was found in diabetic patients with a family history of diabetes, which was consistent with a report that reduction in risk of type 2 diabetes onset by PA in patients with a family history of the condition was four times of those without [27]. A family history of diabetes reflects shared genetic and/or environmental risk factors and their interactions affect, and mechanism involved in may be complex and needed to be further explored. Significant associations were not found in diabetic patients with CVD, and a possible explanation may be the potential health benefits were offset by the impaired cardiac function.

Despite the health benefits of PA for major non-communicable diseases being shown in many studies [28], more than half of the Australian adults failed to adhere to the public health recommendations for PA due to low awareness [29]. Based on the current findings, the risk of DR progression could be reduced by almost 40% with a 14 sessions PA per week, which was comparable no less than 30 minutes PA for five days per week in the guidelines [30]. Education and promotion of the benefits of PA should be included in the comprehensive management of diabetes and its complications.

Strengths of our study include a large sample size with a long follow-up period of 10 years. This is of critical importance for a study on risk factors for DR development and progression because the development of DR would require 5–10 years and the progression to PDR usually would take 10–20 years or even longer, after the diagnosis of diabetes [31]. In addition, the diabetic patients were recruited from the 45 and Up Study, a population-based cohort; therefore the study participants were representative in terms of the severity of diseases, health behavior, and accessibility to care. This is substantially better than studies based on in-hospital patients, where the participants tended to have more severe diseases and have better health awareness.

Some limitations should be illustrated. Firstly, retinal photocoagulation treatment was used as a surrogate for severe DR progression because the retinal photograph was not available for each individual participant. This classification based on treatment instead of diagnosis could lead to some ascertainment bias, for example, those who had no access to treatment could have been missed in the diagnosis. However, this bias was minimal in Australia, where the Medicare coverage is a hundred percent among Australian residents. Severe DR, such as PDR, should already affect vision, and therefore, it is unlikely for a PDR patient to not seek care in Australia. Secondly, PA was assessed based on a self-reported questionnaire, its accuracy could be compromised by recall bias. Although a more objective measure of PA (e.g. accelerometry) which could assess the accurate duration and intensity of PA would be preferable, collecting objective data in such a large study is also often impractical. In consideration of cost-effectiveness of the intervention, self-report measurement remains as the only acceptable method recommended by the WHO for large-scale investigations [32].

## Conclusions

Higher PA level was independently associated with a lower risk of DR progression among working-aged diabetic patients based on a 10-year follow-up of a large cohort of diabetes patients recruited in the community. Further researches exploring specific PA type and quantity using objective measurement for prevention of DR onset and progression are needed to better guide DR care and improve patient outcome.

## Abbreviations

PA: physical activities
DR: diabetic retinopathy
RPC: retinal photocoagulation
HR: hazard ratio
CI: confidence Interval
sNPDR: severe non-proliferative diabetic retinopathy
PDR: proliferative diabetic retinopathy
NSW: New South Wales
MBS: Medicare Benefits Schedule
PBS: Pharmaceutical Benefits Scheme
BMI: body mass index
MET: metabolic equivalent
CVD: cardiovascular disease
